# Effects of the Environmental Temperature on *Aedes aegypti* and *Aedes albopictus* Mosquitoes: A Review

**DOI:** 10.3390/insects9040158

**Published:** 2018-11-06

**Authors:** Joanna M. Reinhold, Claudio R. Lazzari, Chloé Lahondère

**Affiliations:** 1 Department of Biochemistry, Virginia Polytechnic Institute and State University, Blacksburg, VA 24061, USA; reinjm0@vt.edu; 2 Institut de Recherche sur la Biologie de l’Insecte, UMR CNRS 7261, Université de Tours, 37200 Tours, France; claudio.lazzari@univ-tours.fr

**Keywords:** blood-feeding, dispersion, pathogen transmission, gonotrophic cycle, thermotolerance, dengue virus complex, chikungunya virus, Zika virus, West Nile virus, yellow fever virus

## Abstract

The temperature of the environment is one of the most important abiotic factors affecting the life of insects. As poikilotherms, their body temperature is not constant, and they rely on various strategies to minimize the risk of thermal stress. They have been thus able to colonize a large spectrum of habitats. Mosquitoes, such as *Ae. aegypti* and *Ae. albopictus*, vector many pathogens, including dengue, chikungunya, and Zika viruses. The spread of these diseases has become a major global health concern, and it is predicted that climate change will affect the mosquitoes’ distribution, which will allow these insects to bring new pathogens to naïve populations. We synthesize here the current knowledge on the impact of temperature on the mosquito flight activity and host-seeking behavior (1); ecology and dispersion (2); as well as its potential effect on the pathogens themselves and how climate can affect the transmission of some of these pathogens (3).

## 1. Introduction

The environmental temperature (*T_a_*) is one of the most important abiotic factors influencing the physiology, behavior, ecology, and, by extension, the survival of insects [1,2,3]. Indeed, as poikilotherms, the insect internal temperature varies and depends on the temperature of its surrounding environment. Due to local, daily, and seasonal thermal variations, insects have to face risks including desiccation, changes in metabolism, and even losing the ability to move. However, through evolutionary times, insects have developed various strategies to cope with these thermal variations and avoid thermal stress. Whether they synthesize *heat shock proteins*, thermoregulate, or adjust their behavioral activity, insects are able to maintain cellular integrity to optimize their fitness and survival [2,4,5].

For each single task (e.g., flying, feeding), the performance of a given insect species is maximal at a given temperature [3]. However, insects can only perform within a range of temperatures; beyond critical minimum and maximum points, their activity is impossible, and the risk of death increases. Several endogenous factors can affect the range of performance, including the physiological state or the age of the insect. The activity range is species-specific, and some have a large spectrum of temperatures at which they can perform (i.e., thermal generalists) while other species have a much narrower window for maintaining their activity (i.e., thermal specialists) [3,6]. In response to the thermal heterogeneity of their environment, in both time and space, insects have developed several methods of adaptation to protect themselves from thermal stress.

These adaptations have allowed insects to colonize almost all kinds of different habitats. For instance, mosquitoes are found worldwide (except in Antarctica) and can live in a large and eclectic array of ecosystems, from tropical forests to urban areas and tundra. These insects can transmit several important diseases to humans and other animals, including malaria, yellow fever, Zika, dengue, and chikungunya, and are responsible for the death of an estimated one million people each year [7]. Among mosquitoes, two species—*Aedes aegypti* (Linnaeus, 1762), the yellow fever mosquito, and *Aedes albopictus* (Skuse, 1894), the Asian tiger mosquito—have received a lot of attention in the past decade. For these species, integrated vector management, which is a combination of various method, including vector control and education of the public in order to optimize cost, efficiency, and sustainability [8], is extremely important. Indeed, vaccines have not been developed for several of the viruses transmitted by *Ae. aegypti* and *Ae. albopictus*, and vector control remains an essential component of vector management, consisting mainly of space treatment using fogging equipment for these species [9]. Moreover, not only have these species been responsible for the transmission of several pathogens, but their ecological fitting, *sensu* Janzen [10], has made them extremely invasive across the globe.

**Figure 1:**
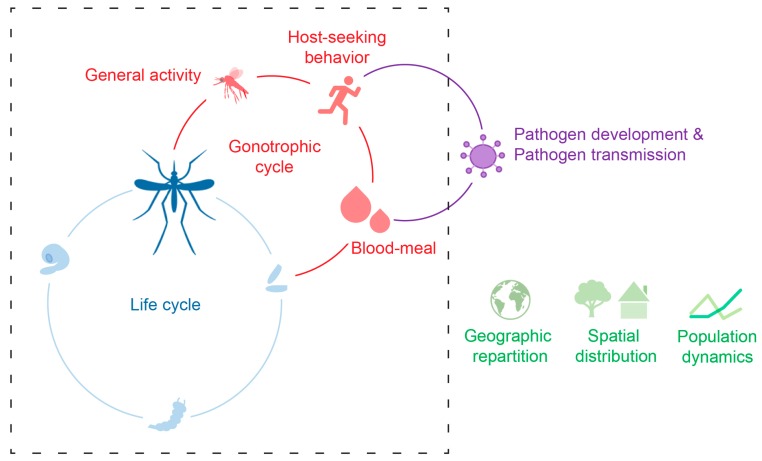
The temperature of the environment (*T_a_*) affects the mosquito development (blue), its activity including host-seeking and blood-meal intake (red), as well as pathogen development and transmission (purple). Consequently, *T_a_* affects species geographic repartition, spatial distribution, and population dynamics (green). The dashed square represents the cycles related to mosquito biology.

In an environmental context of climate change and global warming [11], which is likely to contribute to the spread of these two species, having a better understanding of how the environmental temperature affects mosquito biology appears essential for decrypting the factors driving the ability of these species to invade new areas, where they could potentially transmit pathogens. The present review compiles the current knowledge on the effect of environmental temperature on *Ae. aegypti* and *Ae. albopictus* mosquitoes with a focus on their host-seeking behavior and ecology, including dispersion and vector relevance (Figure 1).

## 2. Fight Activity, Host-Seeking, and Blood-Feeding

*Ae. aegypti* and *Ae. albopictus* are anthropophilic and live in close association with humans, developing preferentially in urban and suburban areas where human hosts are easily available [12,13,14]. Both species are day-biting mosquitoes, exhibiting two main peaks of activity: one in the early morning and the other in the late afternoon [12,13]. *Ae. aegypti* is endophilic (i.e., taking shelter inside houses) and endophagic (i.e., blood-feeding inside houses), but also moves between indoor and outdoor spaces. *Ae. albopictus* also exhibits endophilic activity but is considered exophagic (i.e., most biting occurs outside human dwellings), and is an opportunistic feeder, biting a wide range of hosts, from cold-blooded to warm-blooded animals [13].

Since mosquitoes move from inside to outside (or the opposite) shelters and encounter daily and seasonal thermal fluctuations, they might experience an important range of temperatures that can affect their behavior, including host-seeking. The lower temperature limit for *Ae. aegypti* is around 10 °C, a temperature below which mosquitoes become torpid and unable to move [12,15,16]. Rowley and Graham [17] found that tethered *Ae. aegypti* females were able to sustainably fly between 15 °C and 32 °C, while flight was possible but only for short periods of time at extreme temperatures such as 10 °C and 35 °C. The optimal flight temperature, in terms of duration and distance flown was determined to be at 21 °C, but overall, the flight performance of *Ae. aegypti* tethered females was better below 27 °C. The maximum flight speed (34.1 m/min) was recorded at 32 °C/50% humidity. It is important to highlight that the mosquitoes were able to fly at 10 °C (8.9 ± 0.96 m/min) and at 35 °C (18.2 ± 1.98 m/min). The authors argue that flying at lower temperatures allows the species to be active at the cooler hours of the day (i.e., early morning and late afternoon). Christophers [12] also found that female *Ae. aegypti* wing beat frequency is affected by *T_a_*, measuring 367 beat/s at 18 °C vs. 427 beat/s at 25 °C. Mosquitoes use a wide range of different cues to locate their hosts, including thermal, visual, and chemical information (reviewed in Reference [18]), and host-seeking behavior is tightly linked to their activity (i.e., ability to move and fly). Besides affecting mosquitoes’ general activity and host-seeking behavior, to initiate probing and blood-feeding, a difference between *T_a_* and the host temperature is required [19,20]. Bishop and Gilchrist [21] report a higher percentage of *Ae. aegypti* females imbibing blood at 42 °C when the difference between *T_a_* and the blood-meal was 14 °C (71%) than when the fluid and *T_a_* were at the same temperature (24 °C: 6%; 28 °C: 8%; 37 °C: 19%). The lower temperature limit at which *Ae. aegypti* has been found to cease biting is 15 °C, both in the field and experimentally in the lab [22,23,24]. Connor [22] stated that *Ae. aegypti* is most active at 28 °C. Marchoux et al. [24] found that females fed faster between 26 °C and 35 °C compared to temperatures between 19 °C and 25 °C. The upper temperature limit for blood-feeding is above 36 °C, with the death point being set at 40 °C in *Ae. aegypti* [12]. Interestingly, comparatively less is known regarding the impact of temperature on the flight activity and host-seeking behavior in *Ae. albopictus*.

Unlike numerous other mosquito species, *Ae. aegypti* generally takes several meals during a single gonotrophic cycle [25]. Scott et al. [26] noticed that the frequency of blood intake in this species was positively correlated with *T_a_* (i.e., females fed more often in a warmer environment than in a relatively colder one). However, the authors highlight that this might be due to indirect effects of *T_a_* on the mosquito development, energy storage, and rate of blood-meal digestion and not necessarily due to direct effects on blood-feeding itself [26,27]. Christophers [12] reports that mosquitoes will bite at any temperature at which they are active. In *Ae. albopictus*, it has been shown that the shortest gonotrophic cycle occurred at 30 °C (3.5 days). At this temperature, the authors also recorded the highest number of cycles (3.9) during the female’s life. However, the total number of eggs laid by females during each gonotrophic cycle does not seem to be different at 20 °C, 25 °C, 30 °C, or 35 °C [28]. In this study, *Ae. albopictus* was reared at a constant temperature. Studying the impact of fluctuating temperatures on the development of *Ae. albopictus*, Löwenberg Neto and Navarro-Silva [29] found that when reared under a 27 °C/20 °C thermal regime, the average duration of the females gonotrophic cycle was 11.2 days, with an average of 33.1 eggs laid during the first cycle. In *Ae. aegypti*, Carrington et al. [30] found that the length of the gonotrophic cycle was reduced with increasing mean temperatures, and the shortest was observed at 26 °C and 30 °C. In *Ae. aegypti*, a large Diurnal Temperature Range (DTR) (i.e., the range of *T_a_* that mosquitoes experience over a 24-h period) decreases female fecundity, while a small DTR (8 °C) increases female reproduction [31].

## 3. Ecology and Dispersion

### 3.1. Thermal Optimum and Lower/Upper Zero Developmental and Survival Temperatures

The temperature of the environment alters mosquito population dynamics by affecting the development of the immature stages (i.e., eggs, larvae, and pupae) as well as reproduction (reviewed by Reference [32]). The lower temperature threshold for *Ae. aegypti* to develop is 16 °C, while 34 °C is the upper limit [12]. At a lower temperature (i.e., 8 °C), larvae are motionless and die within a couple of days. Couret et al. [33] also showed that food availability as well as density are factors to consider for affecting the larval development rate and survival in this species, in combination with temperature. The development time from hatching to adult emergence was shorter at higher temperatures (30 °C vs. 21 °C) and correlated with density and food availability. Bar-Zeev [34] found that the time taken by larvae to complete their development was optimal at 32 °C and that mortality was significant at 14 °C and 38 °C. The highest temperature at which development fully occurred was 36 °C. The same author reported 40 °C to be the thermal upper limit for *Ae. aegypti* females, while immature stages were found to survive short exposure to *T_a_* up to 45 °C [35]. *Ae. albopictus* can develop and survive in a wider range of *T_a_*. Indeed, the lower developmental zero temperature is 10.4 °C, with an optimum of 29.7 °C [28]. This species was shown to fully develop between 15 °C and 35 °C and to survive longer at lower temperatures (15 °C vs. 35 °C) in both females and males. It is interesting to mention that different results have been found for this species across studies, depending on the region of origin of the tested populations and their tolerance to cold. For example, Teng and Apperson [36] showed that the larval development temperature threshold was around 9 °C in the same species.

### 3.2. Phenology and Population Dynamics 

*Ae. albopictus* occurs in both temperate and tropical areas and has the ability to overwinter as an egg [37] or an adult. Moreover, in sub-tropical regions, they can also lay eggs that will hatch and not enter diapause [38]. Hawley et al. [39] tested the overwintering survival capacity of different strains of *Ae. aegypti* and *Ae. albopictus* in Indiana (USA). While none of the *Ae. aegypti* strains survived, strains of *Ae. albopictus* acclimated to North American and Asian conditions had a higher survival rate compared to tropical strains. Moreover, within the USA, strains from the south had a lower ability to overwinter than those from northern parts of the country. This highlights the strong capacity of *Ae. albopictus* to adapt rapidly to new thermal conditions and spread to colder regions [40]. *Ae. aegypti* is primarily found in tropical and sub-tropical regions, but it is not completely bound by outside temperatures, being the most cosmopolitan species among insect vectors [41]. In this species, continuous breeding is tightly linked to higher *T_a_*, and these mosquitoes will typically not survive winter as an adult under cold climatic conditions but rather overwinter at the egg stage [12,42]. However, an overwintering population of *Ae. aegypti* adults was recently located near Washington D.C. (USA), which has an average winter temperature lower than what has previously shown to be the limit for the development of this species [43]. The authors argued that *Ae. aegypti* is taking advantage of a subterranean habitat to survive cold temperatures. In tropical regions, both mosquito species can be found year-round, especially in urban areas. Tsunoda et al. [44] reported that the lowest water temperature (i.e., 14 °C) in artificial containers was above the lower thermal limit for *Ae. aegypti* and *Ae. albopictus* development, thus contributing to a yearly occurrence of these mosquitoes and some of the viruses they transmit, such as dengue. Both species’ eggs are resistant to desiccation, allowing them to survive unfavorable conditions and contributing to their ability to spread to new areas via the national and international transportation of materials (e.g., tires, plant pots) containing viable but dormant eggs [41]. Importantly, diurnal and seasonal *T_a_* variations affect the development, density, and dispersion of both species. Carrington et al. [31] used a dynamic model to estimate the thermal limit under which an *Ae. aegypti* mosquito population may persist and found that both small and large DTRs can affect the population dynamics. Seasonal variations in *T_a_* can greatly impact *Ae. aegypti* and large DTR (20 °C) negatively impacts survival [45]. Soper [41] highlights that seasonal fluctuations in terms of range and density are observed, especially at the periphery of the species regional distribution. While *T_a_* has an important effect on the population dynamics, rainfall and drought also impact mosquito density and dispersion in both temperate and tropical regions [46]. In Japan, Suwonkerd et al. [47] found that relatively cooler *T_a_* may explain the decline of *Ae. albopictus* in the dry season. *Ae. aegypti* populations decreased earlier in the season compared to *Ae. albopictus*, but this was not associated with the co-occurrence of the two species at the different sites. Mogi [48] studied several mosquito populations in the same country and highlighted that warmer temperatures are likely to affect overwintering strategies and pathogen transmission by modifying both mosquito biting activity and distribution as well as pathogen development. Beyond responding to changes of thermal conditions, mosquito population dynamics are affected by several other factors including photoperiod (i.e., short days inducing diapause). Tsunoda et al. [49] showed that day length affects egg hatching in *Ae. albopictus* and suggested that under milder (i.e., tropical) conditions, overwintering is not as prevalent in populations in temperate areas.

### 3.3. Spatial Distribution and T_a_ Variability

*Ae. albopictus* is capable of surviving in much colder climates than *Ae. aegypti*, and this species has also adapted well to urban environments. Tsuda et al. [50] showed that *Ae. aegypti* was more commonly found in urban areas while *Ae. albopictus* was found more in rural areas in Thailand, but the pattern of precipitation during their two field seasons was not different between the two sites. If no mention is made regarding *T_a_*, it can be hypothesized that an urban habitat has a higher *T_a_* compared to a rural area, where vegetation is more abundant. *Ae. albopictus* is also mainly exophagic, which means that air conditioning in the summer would not have as much of an effect on it, as it would on *Ae. aegypti* [51]. *Ae. aegypti* can be endophilic and endophagic, allowing this species to reap the benefits of controlled indoor temperatures in extreme heat and cold as well as utilizing man-made structures filled with rain water for oviposition, which allows this species to thrive in urban environments [52]. This highlights that *Ae. albopictus* has developed several strategies to cope with a wider range of *T_a_* and adapt to local thermal conditions. Moreover, it has been shown that environmental variability can actually promote the occurrence of the sudden increased abundance of *Ae. aegypti* [53,54]. With higher *T_a_* in the summer, the yearly pattern of distribution of *Ae. aegypti* in temperate and sub-tropical areas is indeed greatly affected [42]. Applying Schmalhausen’s law [55] to explain both the ecological and evolutionary consequences of a fluctuating environment, in particular in *Ae. albopictus*, Chaves [56] highlighted that perturbations within the normal thermal range have little effect under abnormal or stress conditions, but small perturbations have a stronger effect on individuals. In the same species, Chaves [57] showed that spatial and temporal abundance patterns are most affected by temperature and precipitation, respectively, and that co-occurrence with other species, in this case *Ae. japonicus* and *Ae. flavopictus*, also has major consequences on population dynamics and distribution. Higa et al. [58], in their study on the geographical distribution of these mosquitoes along a north-south transect in Vietnam, showed that *T_a_* affects, in combination with rainfall, the density of both mosquito species. They also mention that in regions where both species were found, climatic conditions are considered milder and habitat heterogeneity becomes a much more important factor explaining spatial distribution.

### 3.4. Vector Distribution in the Context of Climate Change

A recent study by Kraemer et al. [59] reported that the global distribution of both vectors was never considered as important in the past as it is today. *Ae. aegypti* and *Ae. albopictus* have been able to greatly expand their geographical distribution worldwide in the past 30 years, and several studies have shown that climate change is likely to impact the range of these two species [60,61]. For example, Kearney et al. [62] studied the impact of climate change on the distribution and abundance of *Ae. aegypti* in Australia using biophysical models and evolutionary theory and found that water availability, egg desiccation, and tolerance to colder *T_a_* are important factors with the potential to drive the establishment of these mosquitoes in new regions in the south and north of the country. Alto and Juiliano [63] investigated how temperature and precipitation regime affect *Ae. albopictus*. A relatively high *T_a_* (i.e., 30 °C) was more favorable to adult development, both in terms of total number of individuals completing their development and time to adult emergence. This study also showed that in a mild environment (i.e., 22 °C), this species is still able to produce offspring and develop; this could contribute to the colonization of northern (i.e., cooler) regions of the USA. Yang et al. [64] used mathematical modeling (i.e., population dynamics theory) to determine that 29.2 °C was the optimal temperature to produce the highest amount of offspring in *Ae. aegypti* mosquitoes and maintain a viable mosquito population. 

Several studies have also highlighted the impact of environmental conditions on the co-occurrence and inter-species competition between *Ae. aegypti* and *Ae. albopictus*. Since its arrival in the USA, *Ae. albopictus* has led to the decline of *Ae. aegypti* populations in several regions [65]. Lounibos et al. [66] compared the development and survival rates of both species when maintained at different *T_a_*, in combination with other factors including food availability, and found that larval competition outcome was limited between 24 °C and 30 °C and that survival rate was unaffected. Kobayashi et al. [67] used a geographical information system (GIS) and larval survey to analyze the distribution of *Ae. albopictus* in Japan and predict its future expansion both in Japan and the USA. They showed an important correlation between the annual mean temperature and the density of *Ae. albopictus* and revealed that this species is moving further north. They estimated that its current range is also correlated with an average annual *T_a_* of 11 °C in both countries, and that global warming will allow *Ae. albopictus* to expand northward. Mogi and Tuno [68] studied *Ae. albopictus* in its native geographical range using a retrospective approach, including local thermal conditions that have allowed this species to change its pattern of distribution in Japan. They showed that higher *T_a_* during the winter was one important factor that had driven the expansion of the geographical range of *Ae. albopictus*.

Climate change is expected to result in major shifts in vector distribution and/or in the expansion of geographical ranges of both mosquito species with a potential health impact on local populations of humans and other animals due to an enhanced transmission rate of pathogens, including dengue and Zika [46,69,70,71,72]. Rochlin et al. [73] estimated that in the context of climate change, regions with suitable environmental conditions for the development of *Ae. albopictus* will increase by 50% by the end of the century, placing another 30 million people at risk.

## 4. Pathogen Transmission

The feeding and living habits of *Ae. aegypti* and *Ae. albopictus* make these species efficient vectors for human diseases. An arthropod is considered to be a competent vector of an arbovirus or other pathogens if the species demonstrates that it can naturally acquire, be infected by, and transmit a pathogen [74]. This is true for *Ae. aegypti* and *Ae. albopictus*, since they meet these criteria for several arboviruses [75,76]. While *Ae. aegypti* is generally considered a more competent vector for several arboviruses, such as dengue, chikungunya, Zika, yellow fever, and Mayaro, *Ae. albopictus* can vector many of the same viruses with less competence but with the ability to expand its range into colder regions [77]. *Ae. albopictus* also transmits diseases such as West Nile virus, eastern equine encephalitis, and dirofilariasis. The pathogens these species transmit overlap, which could allow some diseases to spread beyond the range of one vector with the help of the other. The ability of these species to vector these pathogens depends on two main factors: whether the pathogen can infect the midgut cells and whether the pathogen can disseminate in order for the vector to transmit it to a new host. Temperature can affect both of these factors, in addition to the development of the mosquito and the time between infection by the pathogen and its transmission (extrinsic incubation period) [57]. The effects of climate change allow these species to develop the pathogens more quickly and spread into new areas, carrying pathogens with them [78,79,80].

### 4.1. Dengue Virus Complex

The dengue virus complex, which refers to the four serotypes that cause dengue fever, has become a major worldwide concern due to its rapid spread and increased intensity in endemic areas [81]. According to the World Health Organization, this is the fastest spreading arbovirus; estimates show that 390 million people are currently infected per year, while 3.9 billion are considered at risk [82,83]. The serotypes can cause different severities of the virus in different people, ranging from asymptomatic to hemorrhagic fever to severe dengue, the latter usually occurring after infection of multiple serotypes, which can lead to coma or death [81]. Vaccines to prevent dengue infections are being developed, with one currently on the market, but only individuals who are seropositive are recommended to receive the vaccine. Because of the limited use of this vaccine, vector control is still the main method to prevent the spread of this virus. Eradication was achieved in the Americas in the 1950’s and 1960’s in an attempt to reduce yellow fever outbreaks, but *Ae. aegypti* returned in the 1970’s, leading to endemic outbreaks of dengue [52]. Since the 1970’s, dengue presence has expanded from nine countries to being endemic in over 100 [81]. 

*Ae. aegypti* is currently the primary vector, though *Ae. albopictus* can also act as a vector, especially in regions where *Ae. aegypti* is not present [75]. Lambrechts et al. [45] showed that a higher DTR (20 °C)—which would be seen in temperate climates in spring and autumn—has a negative effect on the vector competence for dengue and the survival of *Ae. aegypti* compared to a low DTR (10 °C), such as the temperature ranges typically experienced in summer. As discussed previously, temperature has an effect on the blood-feeding habits and survival of *Ae. aegypti* and potentially *Ae. albopictus*, which would both inherently impact pathogen transmission. *Ae. aegypti* is endophilic and endophagic, rendering this species capable of expanding into regions outside its typical temperature range, meaning it can bring dengue along to new regions, making predictions of dengue dispersion a difficult task [52]. The effect of temperature on the development of *Ae. aegypti* was studied by Couret and Benedict [32], who showed that increased temperatures had a positive impact on the rate of development. Global temperature increases would allow for a greater distribution range, as well as an increase in virus transmission. Watts et al. [84] showed that the extrinsic incubation period (EIP)—the amount of time between the mosquito taking an infected blood-meal to transmitting the disease to the next vertebrate host—for DEN2 in *Ae. aegypti* decreases with higher temperatures (32–35 °C) by several days, potentially allowing for faster transmission as one of the consequences of global warming. An experiment was conducted by Rohani et al. [85] for DEN2 and DEN4 serotypes with similar results. *Ae. aegypti* was also shown to be less likely to disseminate dengue at lower temperatures [86].

Because of the ability of *Ae. albopictus* to survive in colder climates, carry pathogens over the winter due to diapause, and have exophagic tendencies, *Ae. albopictus* does have some abilities for transmitting pathogens that *Ae. aegypti* does not [51,75]. Brady et al. [77] highlighted that the longer lifespan of *Ae. albopictus* causes its vector potential to exceed that of *Ae. aegypti.* However, Whitehorn et al. [87] found that *Ae. albopictus* is as susceptible to dengue as *Ae. aegypti* and can even carry a higher viral burden, but DEN2 and DEN4 are not found in high numbers in the saliva, indicating a lower transmission potential for these serotypes. *Ae. albopictus* is not usually found in nature with dengue in areas where *Ae. aegypti* is present, though it has been shown to transmit it in the lab as well as in regions where *Ae. aegypti* is not present. *Ae. albopictus* is thought to mainly be a secondary vector to maintain dengue in rural areas [65]. For this reason, *Ae. albopictus* is considered to be less important as a vector for dengue, as compared to *Ae aegypti*.

Mathematical modeling has shown a clear dependence on seasonal variation of dengue incidence [64]. This seasonality is often assumed to be due to only vector presence, which is typically worse during warm and rainy seasons. However, Thu et al. [88] found that the propagation of dengue within *Ae. aegypti* increased with humidity, with a preferred humidity over 60% and preferred a *T_a_* range of around 24–31 °C. This shows that the amplification of the virus in the mosquito seems to be optimal in conditions that are also favorable to mosquitoes.

### 4.2. Chikungunya Virus

Chikungunya is an alphavirus that is typically found in Africa and Asia, although it has been spreading and has caused outbreaks in over 60 countries in recent years [89]. Though cases of chikungunya are relatively low compared to dengue, outbreaks in naïve populations can be devastating. The disease can be difficult to track, since it is often mistaken for dengue due to their shared symptoms, vectors, and distribution. Both chikungunya and dengue cause flu-like symptoms, such as fever, headache, and nausea, but chikungunya can cause severe joint pain and rarely causes death, with the exception of a recent outbreak in La Réunion [90]. The primary vector is *Ae. aegypti* and the secondary is *Ae. albopictus* for both chikungunya and dengue, which means that both viruses occur in areas where *Ae. aegypti* and/or *Ae. albopictus* are present [89]. It is thought that most of the spread from the original endemic regions in Asia and Africa is caused by *Ae. albopictus*. A major outbreak in La Réunion, an island off the coast of Madagascar, brought chikungunya to the attention of the world in 2005 and 2006. The number of reported cases exceeded 47,000 in a population of about 776,000, which included over 50 cases of materno-neonatal transmission and chikungunya playing a role in the death of over 200 people [90]. These numbers only include those that expressed clinical symptoms, which could only account for as low as 20% of the actual cases [91], and some protective immunity could occur in endemic regions [90]. The first instance of chikungunya in Europe was in Italy in 2007, shortly following the invasion of *Ae. albopictus* [92]. The 2007 outbreak revealed a new strain of chikungunya virus that had adapted to being vectored by *Ae. albopictus*. This mutation (E1-A226 V) gives the virus an advantage for both replication and transmission in *Ae. albopictus* [93]. However, according to Zouache et al. [94], the mutation allowing *Ae. albopictus* to transmit chikungunya may only be advantageous in different environments. This is due to the interaction between the mosquito population, the genetics of the virus, as well as environmental temperature, which means that temperature plays a large role in transmission of chikungunya. The outbreak in 2007 also showed that *Ae. albopictus* has the potential to bring diseases with it to any area to which it can expand [51], and there is a possibility that transovarial and venereal transmission in mosquitoes may lead to a resurgence of the disease [95,96,97]. The first case of chikungunya in the Americas occurred in 2013 on the Caribbean island of St. Martin, and the virus spread to Central and South America and the southern USA in 2014 [98,99]. Local transmission has not been reported in the USA since 2015, but imported cases still occur in several states each year [100]. No vaccine is currently available to prevent chikungunya, so vector control and personal protection from mosquito bites are the main methods for avoiding disease transmission. The spread of the disease over the last decade to new and higher latitudes is at present a cause for concern [89].

### 4.3. Zika Virus

Zika virus also belongs to the family flavivirus and resembles dengue so much that it could almost be referred to as the “fifth dengue serotype”, since serotypes are based on the cross-neutralization of antibodies, and Zika has the potential to neutralize dengue antibodies [101]. Zika is vectored mainly by *Ae. aegypti* in the Americas, as well as other *Aedes* species [102,103], but it can also be transmitted through sexual intercourse and from mother to fetus. The disease has gained interest over the last several years when it was linked to microcephaly and other complications in infants born to mothers who were infected by Zika during pregnancy [102]. Typically, only one out of every five people who are infected with Zika displays symptoms, which include fever, rash, muscle and joint pain, sinus congestion, and other flu-like symptoms [102]. Zika was first seen in humans in Uganda in 1952, and, until 2007, it was primarily endemic in countries of Africa and Asia. The first instance outside of the endemic areas occurred on the Island of Yap [104], and this strain continued to spread from there into French Polynesia, causing a large outbreak in 2013, and moved to Brazil in 2015 [105,106]. Shortly after Zika began spreading in Brazil, large increases in microcephaly in newborns and Guillain-Barré syndrome in adults were observed, being quickly connected to Zika. It has since spread across South America, and autochthonous transmission cases have been reported in more than 80 countries worldwide [102,107]. There is unfortunately no evidence about how Zika progresses in different temperatures, which means the main way to track its possible impact and spread is through understanding its vectors [108]. Though some studies have shown that other *Aedes* species, such as *Ae. vexans*, can vector Zika [103,109,110], there is still little evidence for the vector competence of species other than *Ae. aegypti*, such as *Ae. albopictus*, which could significantly change the possible range of Zika’s impact [111,112]. There is currently no vaccine or treatment for Zika virus, so vector control remains critical [102].

### 4.4. West Nile Virus

West Nile virus is another member of the flavivirus family, though it is more closely related to Japanese encephalitis and St. Louis encephalitis than to dengue and Zika [51]. West Nile virus can be very severe and even fatal to those who display symptoms, though only about 20% of people that are infected will show any signs. The severe symptoms are referred to as West Nile fever, which is a febrile disease with nausea, vomiting, body aches, and headaches, sometimes with a rash. Severe West Nile disease occurs in about 0.67% of those infected and contains the symptoms of West Nile fever with additional neurological problems, which could lead to coma and death [113]. West Nile virus mainly affects humans and horses, with birds and other non-mammals, such as alligators, acting as reservoirs and amplifiers for the disease [113,114]. The virus was first seen in Uganda in 1937 and has been circulating in Africa, the Middle East, Europe, and Australia for years. A strain of West Nile virus from Israel and Tunisia was brought into the USA via New York in 1999 and was picked up by native mosquitoes, causing a large outbreak [115]. The virus is now also considered endemic to North America [113]. West Nile virus can be vectored by several different types of mosquitoes, including species in the genera *Culex* (with *Cx. pipiens* as the main vector), *Ochlerotatus*, and *Aedes*, including *Ae. albopictus* [113,116]. *Ae. albopictus* has been shown to be susceptible to infection and easily disseminated West Nile virus, while *Ae. aegypti* did not show susceptibility [116]. *Ae. albopictus* has also proven to be a natural vector in the wild and could possibly act as a bridge vector from enzootic cycles to urban cycles due to its opportunistic feeding habits [75,117]. Like most arboviruses, seasonality has been observed in West Nile virus, which is in part due to vector density increasing during rainy and warm months, but it is also related to the development of the pathogen in the mosquito. Similar to dengue, infection and dissemination rates (tested in *Cx. pipiens quinquefasciatus*) increased with temperature, recording the lowest rates at 25 °C and the highest at 30 °C [118]. Dohm et al. [119] also found that dissemination rates were less than 30% for mosquitoes tested with an extrinsic incubation temperature of 18 °C, with the highest dissemination rate at 30 °C. These studies showed that relatively higher temperatures positively affect the amplification of West Nile virus in the vector, allowing for higher transmission rates in warmer environments.

### 4.5. Yellow Fever Virus

Yellow fever is another arbovirus in the family of flaviviruses. This virus does not always cause symptoms, but when it does, they can present in two stages. The first stage is moderate, which is characterized by fever, muscle pain, and vomiting, and the second is more severe and includes jaundice, renal failure, and bleeding. The more severe forms may be fatal within about a week. Because there is no cure for the disease, and it can cause such serious cases, it is important to control yellow fever virus as much as possible [120]. Yellow fever virus is now endemic to several countries in Africa and South America, but it caused widespread epidemics when it reached the Americas and Europe in the late 17th century. It circulates in nature in two cycles: a sylvatic in monkeys and an urban cycle in humans. *Ae. aegypti* is the main vector for the urban cycle, and *Ae. albopictus* is thought to bridge the gaps between the sylvatic and urban cycles due to its peri-domestic, opportunistic feeding habits [121]. The existence of the sylvatic cycle makes control of the disease more difficult, as does transovarial vertical transmission in mosquitoes, since it is maintained in different primates and different mosquitoes, such as *Ae. africanus*, *Haemagogus* spp., and *Sabethes* spp. [121,122,123]. Because the disease is maintained outside of the urban cycle, bridge vectors like *Ae. albopictus* can carry the disease back into the urban cycle, which can lead to outbreaks. Yellow fever virus was almost completely eradicated from the Americas in the mid-1920’s, but the disease re-emerged from the sylvatic cycle [124]. There is a highly efficient vaccine available for endemic areas, but this virus can be devastating to naïve populations, and outbreaks can still occur when the coverage of vaccination is not wide or quick enough, or if vector control is not implemented [120,121].

The spread of yellow fever virus is known to be affected by temperature. Davis [125] found that the EIP in *Ae. aegypti* decreased with an increase in temperature. The mosquitoes were able to transmit the disease in as short as four days after infection at 37 °C, while they could not transmit it at 18 °C or less, unless the temperature later increased. These data show that yellow fever virus can be transmitted after a shorter time in warmer weather. Although yellow fever virus has caused epidemics in typically temperate climates, these outbreaks would be seasonal in nature due to the effect temperature has on the virus. It is therefore important to focus on tropical and sub-tropical areas where yellow fever is endemic with strategies for both vector control and widespread use of vaccines.

### 4.6. Other Pathogens

In addition to the above-named viruses, *Ae. aegypti* and *Ae. albopictus* act as vectors for several other diseases. *Ae. albopictus* has been shown to be able to experimentally vector over 20 viruses, though there is no field evidence supporting the natural transmission of all of them [126].

*Aedes albopictus* is a potential bridge vector for eastern equine encephalitis (EEE), which is a virus that affects both humans and horses. EEE has been isolated from *Ae. albopictus* in Florida, USA [127]. Komar et al. [128] found that, when experimentally infecting *Aedes* mosquitoes with EEE, 100% of blood-fed *Ae. albopictus* were infected, compared to 40% of *Ae. aegypti*. Although climate change would have an effect on the distribution of the vector, Chamberlain and Sudia [129] found that temperature had little effect on the development and amplification of the virus in the vector, another *Aedes* species (*Ae. triseriatus*), though lower temperatures caused lower transmission rates and short exposures to high temperatures elongated the incubation period and decreased the rate of transmission.

Mayaro is a virus very similar to chikungunya, which exists in a sylvatic cycle in South America. However, cases have been seen closer to urban areas, and *Ae. aegypti* has been shown to experimentally transmit the virus [51]. A case of a young boy in Haiti who was found to be positive for both Mayaro and dengue showed that Mayaro may have serious public health implications [130]. da Costa Carvalho and Fournier [131] showed that temperature has an effect on the virus when Mayaro virus is subjected to heat stress (37 °C) in *Ae. albopictus* cells, and the virus halts translation of its own proteins, allowing the host cell to synthesize *heat shock proteins*. According to Hotez and Murray [132], Mayaro may have the potential to spread through the Americas, much like dengue and chikungunya.

Some strains of *Ae. albopictus* and *Ae. aegypti* also have the ability to transmit parasitic diseases, such as dirofilariasis and elephantiasis, with *Ae. albopictus* being the more competent vector [75,133,134]. The most common dirofilarial worm transmitted by these mosquitoes is *Dirofilaria immitis*, otherwise known as “heartworm”. This disease can be fatal to canines if left untreated, and immiticide treatments are unpleasant and expensive. The best option is preventing this disease using prophylactic treatments and vector control. Humans can also be infected with dirofilaria, which is mainly endemic to southern Europe, but is spreading north [135]. The seasonality for dirofilariasis, as well as its geographic range, is expanding due to rising global temperatures, allowing for a longer transmission season [136]. Ledesma and Harrington [137] also show that *D. immitis* L3s are significantly affected by temperature fluctuations during the EIP, developing more quickly under fluctuating temperatures than constant temperature within a certain range. Dirofilariids show little preference for vector species compared to some other pathogens, meaning that these worms could be easily introduced to a new region if a competent vector is present. The presence of invasive competent vectors, such as *Ae. albopictus*, in endemic areas can increase the transmission of the disease [138]. Therefore, it is important to understand to what extent changing temperatures would allow the expansion of *Ae. albopictus* and *Ae. aegypti* distribution range, in order to control for this and other diseases.

## 5. Conclusions, Future Directions and Knowledge Gaps

As *Ae. albopictus* and *Ae. aegypti* continue to expand their distribution range, and in the context of global warming, it appears more important than ever to have a good understanding of their biology and ecology. If much is known about the effects of *T_a_* on *Ae. aegypti*, comparatively less information is available for *Ae. albopictus*. Focusing on this knowledge gap, in particular regarding the effects on the activity and host-seeking behavior of this species of mosquito, is however critical to implement accurate data in population dynamics models to determine future distribution of this disease vector species. Extensive work has been conducted in *Anopheles* mosquitoes and the pathogens they transmit, including *Plasmodium falciparum*, the agent of malaria (e.g., [139,140,141]). However, little is known regarding the effects of temperature on several viruses including chikungunya and Zika viruses (independent of vector or within vector), and these data would allow predictions to be more precise and realistic. It would also be important to know the vectors of diseases, especially emerging infectious diseases like Zika, in order to understand the potential range and build accurate models [111]. More information becoming available for these mosquitoes and the pathogens they vector will allow for a better understanding of what can be done to prevent the spread of disease.
